# An activity-dependent computational model of development of the retinotopic map along the dorsoventral axis in the primary visual cortex

**DOI:** 10.1186/1471-2202-15-S1-P189

**Published:** 2014-07-21

**Authors:** Ryan Thomas Philips, V Srinivasa Chakravarthy

**Affiliations:** 1 Department of Biotechnology, Indian Institute of Technology Madras, Chennai 600036, Tamil Nadu, India

## 

Primate vision research has shown that in the retinotopic map of the primary visual cortex, eccentricity and meridional angle are mapped onto two orthogonal axes [1]: whereas the eccentricity is mapped onto the nasotemporal axis, the meridional angle is mapped onto the dorsoventral axis. Such a map has been approximated by a complex log map [1]. While the development of the map along the nasotemporal axis is controlled by a combination of EphA-ephrin-A signaling as well as spontaneous retinal waves; the mechanisms involved in the map formation along the dorsoventral axis are still unknown [2]. Neural models with correlational learning have successfully explained other visual maps like orientation maps and ocular-dominance maps. No such network models of retinotopic map development exist. In this paper we propose an activity based model which simulates the large-scale development of the retinotopic map along the dorsoventral axis. The architecture consists of a LISSOM (Laterally Interconnected Synergetically Self Organizing Map) [3] with 2 layers; representing the retina, and the V1 respectively (see Figure 1A). At each time step, each neuron in V1, combines the afferent activation (*ζ_r1,r2_*) along with its lateral excitations and inhibitions (*η_kl_*) from the previous time step.

**Figure 1:**
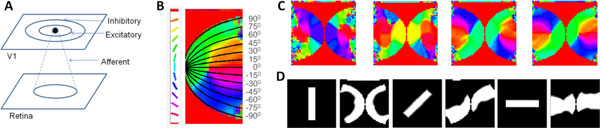
**(A) Schematic representation of the LISSOM architecture**. **(B) **V1 retinotopic map developed after training, superimposed with the complex logarithmic map for verification. **(C) **V1 map development at 200, 400, 600, 750 iterations. **(D) **Input (Retina) & Output (V1) pairs, post training; for 90^°^, 45^°^, 0^° ^bars given as input.

ηij(t)=σ(∑r1,r2ζr1,r2 μij,r1r2+ γE ∑k,lEij,kl ηkl(t-1)-γI ∑k,lIij,kl ηkl(t-1))

The afferent (*μ_ij,r1r2_*), lateral excitatory (*E_ij,kl_*) and lateral inhibitory (*I_ij,kl_*) weights adapt based on a normalized Hebbian mechanism. The input to the retinal layer consists of rectangular bars of varying dilation and rotation; since images on the retina could be considered as different projective transforms of the objects seen. The outer boundary of the V1 layer is also constrained to simulate the flattened V1 surface area (see Figure 1B). After training for 750 iterations (see Figure 1C), it may be observed in the developed map that eccentricity is mapped along the x-axis while the meridional angle is mapped along the y-axis, an organization that bears strong resemblance to the complex logarithmic map [1] (see Figure 1B, Figure 1D).

## Conclusions

A neural activity based model for the development of retinotopic map along the dorsoventral axis is demonstrated and the final map developed is compared with experimental results approximated by the complex log map equations.
